# Correlation between modified LEMON score and intubation difficulty in adult trauma patients undergoing emergency surgery

**DOI:** 10.1186/s13017-018-0195-0

**Published:** 2018-07-24

**Authors:** Sung-Mi Ji, Eun-Jin Moon, Tae-Jun Kim, Jae-Woo Yi, Hyungseok Seo, Bong-Jae Lee

**Affiliations:** 1 Department of Anesthesiology and Pain Medicine, College of Medicine, Dankook University, Cheonan, South Korea; 2 Department of Anesthesiology and Pain Medicine, Kyung Hee University Hospital at Gangdong, College of Medicine, Kyung Hee University, Seoul, 05278 South Korea

**Keywords:** Airway, Difficult intubation, Emergency surgery, LEMON score, Trauma

## Abstract

**Background:**

Prediction of difficult airway is critical in the airway management of trauma patients**.** A LEMON method which consists of following assessments; Look-Evaluate-Mallampati-Obstruction-Neck mobility is a fast and easy technique to evaluate patients’ airways in the emergency situation. And a modified LEMON method, which excludes the Mallampati classification from the original LEMON score, also can be used clinically. We investigated the relationship between modified LEMON score and intubation difficulty score in adult trauma patients undergoing emergency surgery.

**Methods:**

We retrospectively reviewed electronic medical records of 114 adult trauma patients who underwent emergency surgery under general anesthesia. All patients’ airways were evaluated according to the modified LEMON method before anesthesia induction and after tracheal intubation; the intubating doctor self-reported the intubation difficulty scale (IDS) score. A difficult intubation group was defined as patients who had IDS scores > 5**.**

**Results:**

The modified LEMON score was significantly correlated with the IDS score (*P* < 0.001). The difficult intubation group showed higher modified LEMON score than the non-difficult intubation group (3 [2-5] vs. 2 [1-3], respectively, *P* = 0.017). Limited neck mobility was the only independent predictor of intubation difficulty (odds ratio, 6.15; *P* = 0.002).

**Conclusion:**

The modified LEMON score is correlated with difficult intubation in adult trauma patients undergoing emergency surgery.

## Background

Successful airway securement by an experienced physician is crucial in the management of trauma patient [1]. However, compared with other types of patients requiring tracheal intubation, trauma patients have a higher risk of intubation difficulty [2]. In trauma patients requiring emergency surgery, there may not have enough time to evaluate patient’s airway, thereby being an increased risk of the unanticipated difficult airway. Furthermore, because of the limited number of advanced airway securing devices or experienced staffs, such a situation that some devices or staffs are unavailable temporarily can be possible. Thus, prediction of the difficult airway and preparing appropriate device or staffs is critical in the airway management of trauma patients.

Conventional tools for predicting difficult airway, such as the Mallampati score, have a limited application in trauma patients [3]. The LEMON method, which consists of following assessments: Look-Evaluate-Mallampati-Obstruction-Neck mobility, can be used to predict difficult intubation in the emergency setting [4], and the modified LEMON score (also called “LEON” score), which excludes the Mallampati classification from the original LEMON score, has been developed for the identification of difficult airways [5].

In the present study, we retrospectively investigated the ability of the LEON score to predict intubation difficulty by assessing the correlation between the LEON score and intubation difficulty score in adult trauma patients undergoing emergency surgery.

## Methods

### Patients

After the approval of the institutional review board (approval number: 2016-11-014), electronic medical records of adult trauma patients who underwent emergency surgery under general anesthesia in an operating theater between March 2016 and August 2016 were reviewed retrospectively. Patients who were already intubated before anesthesia induction or underwent surgical procedures under regional anesthesia were excluded.

### Data collection

**Fig. 1:**
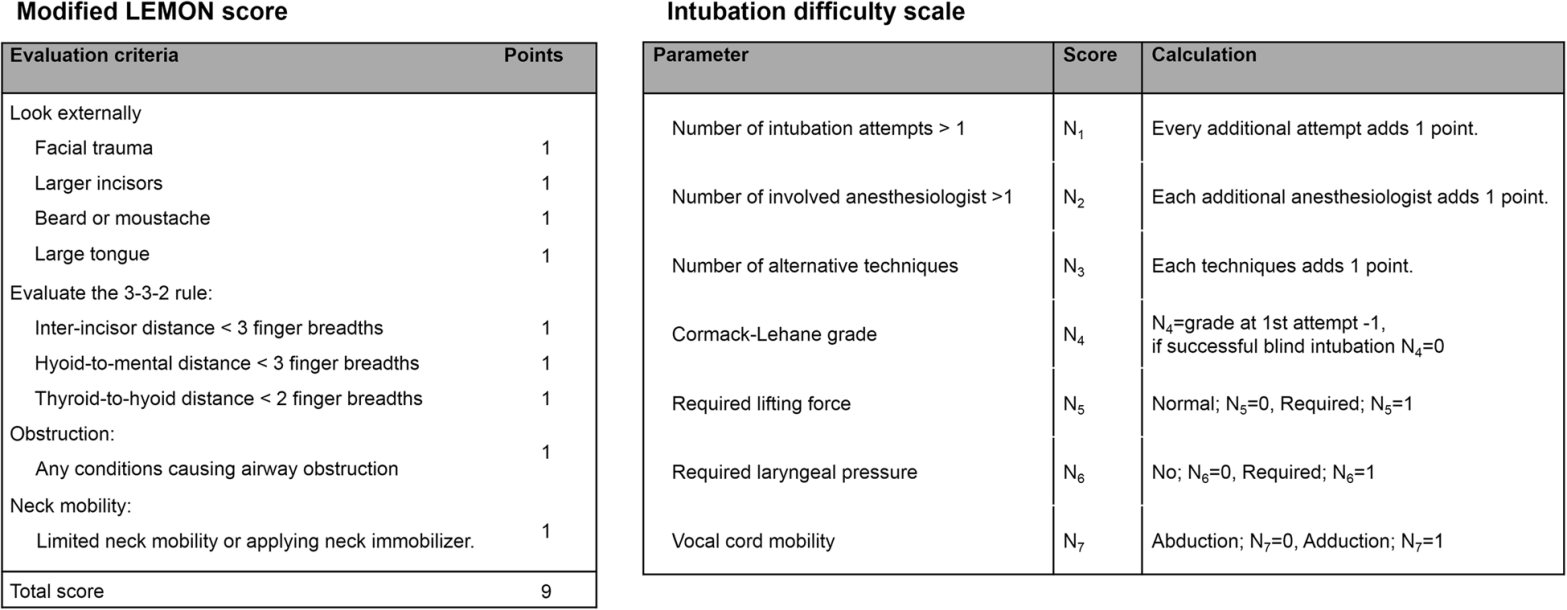
Modified LEMON (LEON) score and intubation difficulty scale score

All patients’ airways were evaluated by the well-trained residents or attending members of staff of the anesthesia department before anesthesia induction. Patient’s airway assessment was performed according to the LEON method (Fig. 1, left) as follows: (1) Look, look at the patient externally for characteristics that are known to cause difficult laryngoscopy, intubation, or ventilation—in the LEON method, “Look” criteria assesses for presence of four features (facial trauma, large incisors, beard or mustaches, and large tongue); (2) evaluate the 3-3-2 rule—assess the alignment of the pharyngeal, laryngeal, and oral axes; (3) obstruction—presence of any conditions that can cause airway obstruction; and (4) neck mobility—assess for the presence of limited neck mobility or use of a hard neck collar immobilizer. General anesthesia was performed according to the institution’s routine clinical practice. Anesthesia was induced with propofol (1 to 2 mg/kg) and maintained with volatile anesthetics, such as sevoflurane or desflurane. Target-controlled infusions of propofol and remifentanil were used. A neuromuscular blocking agent (NMBA), rocuronium bromide (0.6 mg/kg), was administered to facilitate tracheal intubation. Initially, tracheal intubation was performed by using a direct laryngoscope or video laryngoscope based on the intubating doctor’s choice; however, a lightwand device or fiberoptic bronchoscopy was also used in cases of failed first attempt or difficult intubation. After tracheal intubation, the intubating doctor self-reported in the electronic medical records using the intubation difficulty scale (IDS; Fig. 1, right) as follows: N_1_, the number of supplementary intubation attempts; N_2_, the number of supplementary operators; N_3_, the number of alternative intubation techniques used; N_4_, glottic exposure as defined by the Cormack and Lehane grade (grade 1, N_4_ = 0; grade 2, N_4_ = 1; grade 3, N_4_ = 2; grade 4, N_4_ = 3); N_5_, the lifting force applied during laryngoscopy (N_5_ = 1 if a subjectively increased lifting force was required); N_6_, external laryngeal pressure to improve glottic exposure (N_6_ = 1 if external laryngeal pressure was required); and N_7_, position of the vocal cords at intubation (N_7_ = 0 if vocal cords in abduction or were not visualized; N_7_ = 1 if vocal cords in adduction or blocking the tube passage). The IDS score is the sum of N_1_ through N_7_. An IDS score between 1 and 5 represents slight difficulty, and IDS score > 5 represents moderate to major difficulty. In the present study, patients were divided into the difficult intubation group (group D) and non-difficult intubation group (group ND) according to whether their IDS score was > 5 or ≤ 5.

### Statistical analysis

All analyses were performed using MedCalc® 16.2 (MedCalc Software, Ostend, Belgium). Continuous variables were compared using the Student’s *t* test or Mann-Whitney *U* test. Categorical variables were analyzed using a chi-square test, and the Cochran-Armitage test was used for trend analysis. Correlation between the LEON score and IDS was calculated using the Spearman’s rank correlation. To determine the relationship between one dependent factor and one or more independent factors, a logistic regression analysis was performed. Data are expressed as means ± standard deviations, medians [interquartile ranges], or number (%). *P* < 0.05 was considered to be statistically significant.

## Results

During study period, a total of 114 cases were reviewed. Patients’ characteristics are shown in Table 1. There were no differences with respect to the demographic data and type of injury between group D and group ND. There was no patient who had unsuccessful intubation. Direct laryngoscope, video laryngoscope, lightwand device, and fiberoptic bronchoscope were used in 96, 17, 5, and 5 patients (84, 15, 4, and 4%, respectively).

**Table 1:** Patients’ characteristics

Variables	Overall	Group ND (*n* = 96)	Group D (*n* = 18)	*P*
Age (year)	53 [38–61]	52 [18–83]	56 [19–84]	0.492
Sex (male %)	87 (76.3%)	73 (76.0%)	14 (77.8%)	0.874
Height (cm)	168.0 [162.8–175.0]	168.5 [163.0–175.0]	168.0 [159.5–174.3]	0.969
Weight (kg)	68.5 [57.0–75.0]	68.0 [57.0–75.0]	70.0 [57.3–75.8]	0.676
BMI (kg/m^2^)	23.4 [21.1–25.1]	23.3 [21.0–25.1]	24.0 [22.3–25.7]	0.616
Type of injury				
Head and neck	34 (29.8%)	28 (29.2%)	6 (33.3%)	0.728
Chest	7 (6.1%)	6 (6.2%)	1 (5.6%)	0.923
Abdomen	7 (6.1%)	5 (5.2%)	2 (11.1%)	0.341
Spine	10 (8.8%)	8 (8.3%)	2 (11.1%)	0.701
Pelvis	4 (3.5%)	4 (4.2%)	0 (0%)	0.378
Extremities	52 (45.6%)	45 (46.9%)	7 (38.9%)	0.534

Data are expressed as medians [IQRs] or numbers (%)

Group ND: patients show intubation difficulty score ≤ 4

Group D: patients show intubation difficulty score > 5

Head and neck injury includes trauma to the head, facial area, and cervical spine

Spine injury indicates injury of thoracic and lumbar vertebra with/without neurologic complication

*BMI* body mass index

The LEON score was significantly correlated with the IDS score (Spearman’s correlation coefficient: 0.333, *P* < 0.001). The IDS score was 6 [6, 7] in group D, and it was 1 [0–3] in group ND (*P* < 0.001). The Cormack-Lehane grade was significantly higher in group D than in group ND (3 [3, 4] vs. 1 [1, 2], *P* < 0.001). The number of intubation attempts was higher in group D than in group ND (2 [1, 2] vs. 1 [1], *P* < 0.001). The median intubation time was also longer in group D than in group ND (50 [27–80] vs. 17 [13–25] seconds, *P* < 0.001).

**Fig. 2:**
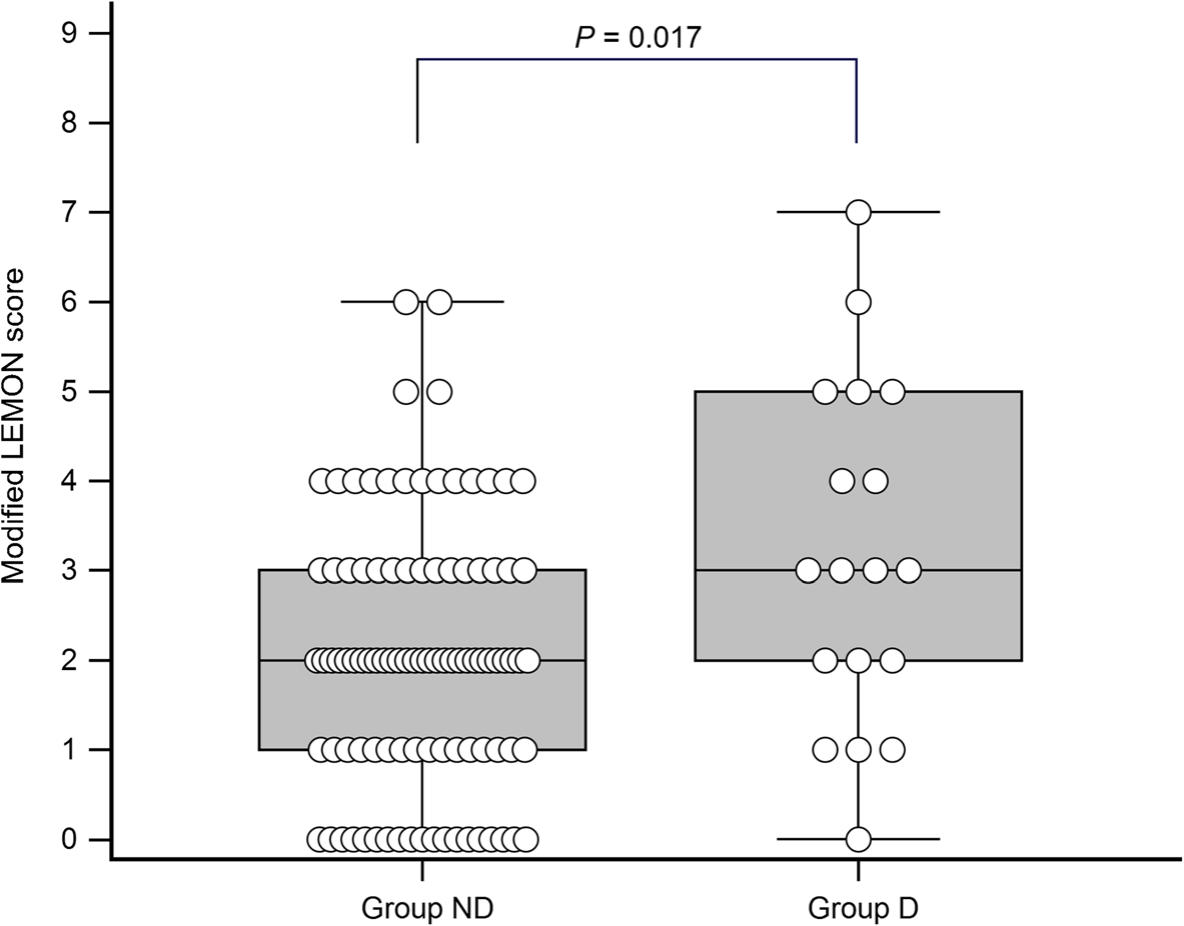
Comparison of the modified LEMON (LEON) scores between group ND and group D. Patients in group ND shows intubation difficulty score ≤ 4 and patients in group D shows > 5. The modified LEMON score was 2 [1–3] in group ND and 3 [2–5] in group D (*P* = 0.017)

The LEON score was higher in group D than in group ND (3 [2–5] vs. 2 [1–3], *P* = 0.017) (Fig. 2). The incidence of difficult intubation tended to increase as the LEON score increased (*P* = 0.005). The logistic regression analysis showed that the LEON score showed a significant correlation with intubation difficulty (odds ratio, 1.55; 95% confidence interval, 1.12–2.14, *P* = 0.008). Among the variables in the LEON score, limited neck mobility was the only independent predictor of intubation difficulty (odds ratio, 6.15: 95% confidence interval, 1.909–19.819; *P* = 0.002) (Table 2).

**Table 2:** The incidence of each variables of modified LEMON (LEON) score and the correlation of each variables with the intubation difficulty

Variables	Overall	Group ND (*n* = 96)	Group D (*n* = 18)	Odds ratio	95% Confidence interval	*P*
Facial trauma, *n* (%)	32 (28.1%)	27 (281%)	5 (27.8%)	0.983	0.320–3.022	0.976
Large incisors, *n* (%)	9 (7.9%)	6 (6.2%)	3 (16.7%)	3.000	0.676–13.309	0.148
Beard or mustache, *n* (%)	17 (14.9%)	12 (12.5%)	5 (27.8%)	2.692	0.814–8.900	0.105
Large tongue, *n* (%)	16 (14.0%)	11 (11.5%)	5 (27.8%)	2.972	0.888–9.943	0.077
Inter-incisor distance < 3 FBs, *n* (%)	49 (43.0%)	39 (40.6%)	10 (55.6%)	1.827	0.662–5.041	0.245
Hyoid-to-mental distance < 3 FBs, *n* (%)	33 (28.9%)	28 (29.2%)	5 (27.8%)	0.934	0.304–2.867	0.905
Thyroid-to-hyoid distance < 2 FBs, *n* (%)	48 (42.1%)	39 (40.6%)	9 (50.0%)	1.462	0.533–4.012	0.461
Obstruction signs, *n* (%)	30 (26.3%)	22 (22.9%)	8 (44.4%)	2.691	0.947–7.647	0.063
Limited neck mobility, *n* (%)	16 (14.0%)	9 (9.4%)	7 (38.9%)^a^	6.152	1.909–19.821	0.002

^a^*P* = 0.001 compared with group ND

Group ND indicates patients show intubation difficulty score ≤ 4 and group D indicates patients show intubation difficulty score > 5

*P* value indicates the significance of univariable logistic regression between each variables and intubation difficulty

Obstruction signs indicate the presence of any conditions such as epiglottitis, peritonsillar abscess, sleep apnea, or upper airway trauma

*FB* finger breadth

## Discussion

In the present study, we found that the LEON score correlated with the intubation difficulty and a LEON score of ≥3 could predict intubation difficulty in trauma patients.

Airway management is a challenging issue in adult trauma patients. Trauma patients may present with a variety of airway difficulties, ranging from promptly recognized to unanticipated difficult airways [6]. Moreover, most of trauma patients requiring emergency surgery do not have adequate time to undergo full preoperative airway evaluation; thus, they can be at an increased risk of unanticipated difficult airway. [4, 7] Therefore, it is crucial to be able to conduct a prompt assessment of the airway and predict difficult intubation. The LEMON score has been effectively used in emergency departments to predict difficult intubation because it can be determined based on the patient’s appearance and observer’s fingerbreadth, and it does not require special cut-off values or additional measurement tools [5, 8]. The Mallampati score component in the LEMON score is difficult to assess in trauma patient [7]; therefore, the LEON score, which excludes the Mallampati score, has been used effectively in clinical situations [5, 9]. Although the LEON score has been validated and widely used in emergency departments, the definitions of difficult intubation in previous studies were ambiguous (i.e., difficult tracheal intubation: tracheal intubation that requires multiple attempts in the presence or absence of tracheal pathology). In the present study, we used the IDS and defined a difficult intubation as IDS score > 5 [2, 10]. By using the IDS, the severity of difficult intubation could be quantified; this enabled an analysis of the correlation between LEON score and intubation difficulty, which can suggest a cut-off value of LEON score in predicting difficult intubation.

It has been previously well-validated that the LEON score can effectively predict difficult intubation in the emergency department [9]. However, comparing with previous report that showed the median value of the LEON score was 1 in both the easy and difficult intubation group [5], our results showed that the median value of the LEON score was 3 in the difficult intubation group and 2 in the non-difficult intubation group. Moreover, our results showed that limited neck mobility is an independent predictor of difficult intubation; this is in contrast with the results of a previous study that showed the thyroid-to-hyoid distance was not an independent predictor of difficult intubation. We thought that this difference may be because of our study population, particularly the high proportion of patients with head and neck injury. Head and neck injury is frequently associated with cervical spine injury [11, 12], and neck immobilization should be considered even without a definite cervical spine injury [13]. The point in the criteria of limited neck mobility may have contributed to a higher LEON score in this study than that reported in the previous study. Moreover, limited neck mobility, which was found to be an independent predictor of difficult intubation in the present study, may also provide evidence supporting the importance of neck mobility in the airway management of trauma patients. The thyroid-to-hyoid distance, despite its clinical significance in a previous study [5], did not significantly contribute the difficult intubation in this study, and the use of video laryngoscope may have affected this observation. Compared with direct laryngoscope, a video laryngoscope can provide an extended view in the vertical plane, which offers an advantage in cases of an anteriorly placed larynx [14]. A video laryngoscope was used for the initial intubation attempt in 15% of the subjects, which may be large enough to attenuate the influence of the thyroid-to-hyoid distance in tracheal intubation.

This study has several limitations. First, the study was conducted with a retrospective design via analysis of electronic medical records. Although the intubating doctor recorded the intubation information right after the induction of anesthesia, the data may have been unreliable because they were self-reported data. Moreover, because the initial intubation device can be selected by intubator’s choice, a selection bias may affect the result. A relatively small population for a retrospective study also contributes to the result. Second, the results of the present study do not reflect patients with very severe and complex traumatic injuries that require immediate airway access. Because we evaluated patients undergoing emergency surgery in the operating theater, patients who underwent immediate tracheal intubation in the emergency department were not included. Third, we used NMBA to facilitate tracheal intubation in the present study. Since NMBA can provide the relaxation of soft tissues and comfortable condition without patient movements, the IDS score can be differed in clinical situations of tracheal intubation without NMBA.

## Conclusion

The LEON score may be used as one of the evidence predicting difficult airway, thereby being helpful to increase safety in the airway management of adult trauma patients undergoing emergency surgery. A patient with LEON score ≥ 3 may have the possibility of difficult intubation, and even in using video laryngoscopy, the limited neck mobility may contribute to the intubation difficulty.

## Abbreviation

IDS: Intubation difficulty scale
NMBA: Neuromuscular blocking agent
